# Investigating the role of AA9 LPMOs in enzymatic hydrolysis of differentially steam-pretreated spruce

**DOI:** 10.1186/s13068-023-02316-0

**Published:** 2023-04-19

**Authors:** Fabio Caputo, Monika Tõlgo, Polina Naidjonoka, Kristian B. R. M. Krogh, Vera Novy, Lisbeth Olsson

**Affiliations:** 1 Division of Industrial Biotechnology, Department of Biology and Biological Engineering, Chalmers University of Technology, Kemivägen 10, 412 96 Gothenburg, Sweden; 2 Wallenberg Wood Science Center, Chalmers University of Technology, Kemigården 4, 412 96 Gothenburg, Sweden; 3 Division of Materials Physics, Department of Physics, Chalmers University of Technology, Kemigården 1, 412 96 Gothenburg, Sweden; 4 Novozymes A/S, Biologiens Vej 2, 2800 Kgs. Lyngby, Denmark

**Keywords:** Biorefinery, Lignocellulose, Softwood, Enzymatic saccharification, STEX, LPMO, *Thermoascus aurantiacus*, Celluclast, Cellic CTec2

## Abstract

**Background:**

To realize the full potential of softwood-based forest biorefineries, the bottlenecks of enzymatic saccharification of softwood need to be better understood. Here, we investigated the potential of lytic polysaccharide monooxygenases (LPMO9s) in softwood saccharification. Norway spruce was steam-pretreated at three different severities, leading to varying hemicellulose retention, lignin condensation, and cellulose ultrastructure. Hydrolyzability of the three substrates was assessed after pretreatment and after an additional knife-milling step, comparing the efficiency of cellulolytic Celluclast + Novozym 188 and LPMO-containing Cellic CTec2 cocktails. The role of *Thermoascus aurantiacus Ta*LPMO9 in saccharification was assessed through time-course analysis of sugar release and accumulation of oxidized sugars, as well as wide-angle X-ray scattering analysis of cellulose ultrastructural changes.

**Results:**

Glucose yield was 6% (*w*/*w*) with the mildest pretreatment (steam pretreatment at 210 °C without catalyst) and 66% (*w*/*w*) with the harshest (steam pretreatment at 210 °C with 3%(*w*/*w*) SO_2_) when using Celluclast + Novozym 188. Surprisingly, the yield was lower with all substrates when Cellic CTec2 was used. Therefore, the conditions for optimal LPMO activity were tested and it was found that enough O_2_ was present over the headspace and that the reducing power of the lignin of all three substrates was sufficient for the LPMOs in Cellic CTec2 to be active. Supplementation of Celluclast + Novozym 188 with *Ta*LPMO9 increased the conversion of glucan by 1.6-fold and xylan by 1.5-fold, which was evident primarily in the later stages of saccharification (24–72 h). Improved glucan conversion could be explained by drastically reduced cellulose crystallinity of spruce substrates upon *Ta*LPMO9 supplementation.

**Conclusion:**

Our study demonstrated that LPMO addition to hydrolytic enzymes improves the release of glucose and xylose from steam-pretreated softwood substrates. Furthermore, softwood lignin provides enough reducing power for LPMOs, irrespective of pretreatment severity. These results provided new insights into the potential role of LPMOs in saccharification of industrially relevant softwood substrates.

**Supplementary Information:**

The online version contains supplementary material available at 10.1186/s13068-023-02316-0.

## Background

Biorefineries that utilize lignocellulosic biomass as feedstock can support the sustainable production of various chemicals and fuels. The most abundant woody biomass in boreal forests, and hence the largest available feedstock for biorefineries in Sweden, is Norway spruce (*Picea abies*). However, spruce and softwoods in general, are highly recalcitrant to enzymatic saccharification than many other biomasses. Softwood recalcitrance is affected by substrate-related factors, such as the S/G ratio of lignin [1] and the hemicellulose composition (consisting of arabinoglucuronoxylan and galactoglucomannan [2, 3]), as well as by more enzyme-related parameters. A key factor when degrading lignocellulosic material is cellulose accessibility [4], defined by the ease with which an enzyme can access its reaction site [5]. Accessibility is determined by the extent with which lignin and hemicellulose cover the cellulose macrofibrils/fibers [3], as well as the ratio between highly ordered (crystalline) and poorly ordered (para-crystalline) cellulose microfibrils [6, 7].

Steam pretreatment (STEX), a mature, scalable, and feedstock-adjustable technology, augments enzyme accessibility and hydrolyzability of softwood substrates [8]. To compensate for the low organic acid content in softwood, an acid catalyst is usually added to break up the lignin–hemicellulosic composite and increase accessibility [8, 9]. Harsh STEX conditions lead to degradation of hemicelluloses and formation of secondary decomposition compounds, thus decreasing the achievable sugar yield and potentially inhibiting microbial fermentation [10–12]. Although milder conditions have been shown to lead to hemicellulose retention [8], strategies to overcome the decreased accessibility and hydrolyzability that follow mild pretreatment conditions [13] must be implemented. One way to boost saccharification efficiency of pretreated material is through an additional milling step, which increases the specific surface area and facilitates enzyme accessibility [14, 15]. However, because milling is energy demanding, its industrial implementation remains limited [16, 17].

In recent years, studies on the role and interplay of hemicellulolytic and cellulolytic enzymes [4, 18, 19] have resulted in improved commercial enzyme cocktails. For example, Celluclast, which possesses mainly cellulose-degrading activity, has been upgraded to Cellic CTec2, which is enriched in hemicellulolytic and accessory activities. The discovery of lytic polysaccharide monooxygenases (LPMOs) of the AA9 family (LPMO9s), which are included in Cellic Ctec2 [20] but in minor amounts in Celluclast [21, 22], paved the way for new generation cocktails for lignocellulosic substrates. In contrast to cellulolytic hydrolases, cellulose-active LPMOs are mono-copper enzymes with a relatively flat active site that uses O_2_ and/or H_2_O_2_ as co-substrates. LPMOs cleave their substrates by oxidizing C1 or C4 carbons, or both [23–26]. As the Cu(II) core must be reduced to Cu(I), reaction conditions for LPMOs must be tailored carefully with respect to reductant, co-substrate, and potential auto-inactivation processes [27–29].

LPMOs attack crystalline cellulose to create binding sites for hydrolytic cellulases [18, 25], although they have been found to process also para-crystalline cellulose [30–32], hemicelluloses [32], and cello-oligosaccharides [28, 33, 34]. Addition of LPMO9s to commercial enzyme cocktails boost the saccharification of several lignocellulose substrates, such as sulfite-pulped spruce [35] and steam-pretreated birch [22, 36]. Evidence that lignin can directly reduce LPMOs, makes them of further appeal in commercial biorefinery concepts because it eliminates the need for additional reductants [22, 37, 38]. However, evidence of LPMO activity on non-model substrates such as mildly steam-pretreated softwood, is still scarce.

The present study aimed to investigate the contribution of LPMOs to the saccharification of three differently steam-pretreated spruce substrates, possessing or not hemicellulose. As a base-case, the three materials were hydrolyzed after pretreatment and after an additionally milling step, using the cellulolytic Celluclast + Novozym 188 and the LPMO-containing Cellic CTec2 cocktails. After assessing that reaction conditions supported LPMO activity, the contribution of the well-studied *Thermoascus aurantiacus Ta*LPMOs to cellulose saccharification in softwood by Celluclast + Novozym 188 was assessed by hydrolysis time courses. Changes to cellulose ultrastructure were evaluated by wide-angle X-ray scattering (WAXS) before and after enzymatic hydrolysis. The obtained knowledge on the potential of LPMOs can be applied to increase carbohydrate conversion during saccharification of industrially relevant softwood substrates.

## Results and discussion

The overall aim of this study was to investigate the role of LPMO9 in the saccharification of softwood substrates. Spruce material was first steam-pretreated under three various conditions to yield solids with or without hemicellulose retention. The three STEX-pretreated spruce materials were compositionally analyzed, and the hydrolyzability evaluated using the cellulolytic enzyme cocktail Celluclast + Novozym188. The materials were additionally milled to potentially increase saccharification, and then hydrolyzed with Celluclast + Novozym188 or the LPMO-containing Cellic CTec2. After verifying that the chosen reaction conditions support LPMO9 activity, Celluclast + Novozym188 was supplemented with *Ta*LPMO9 and the sugar release, accumulation of oxidized sugars, and cellulose ultrastructure were investigated for all three softwood substrates.

### Composition and hydrolyzability of differentially steam-pretreated spruce

**Fig. 1:**
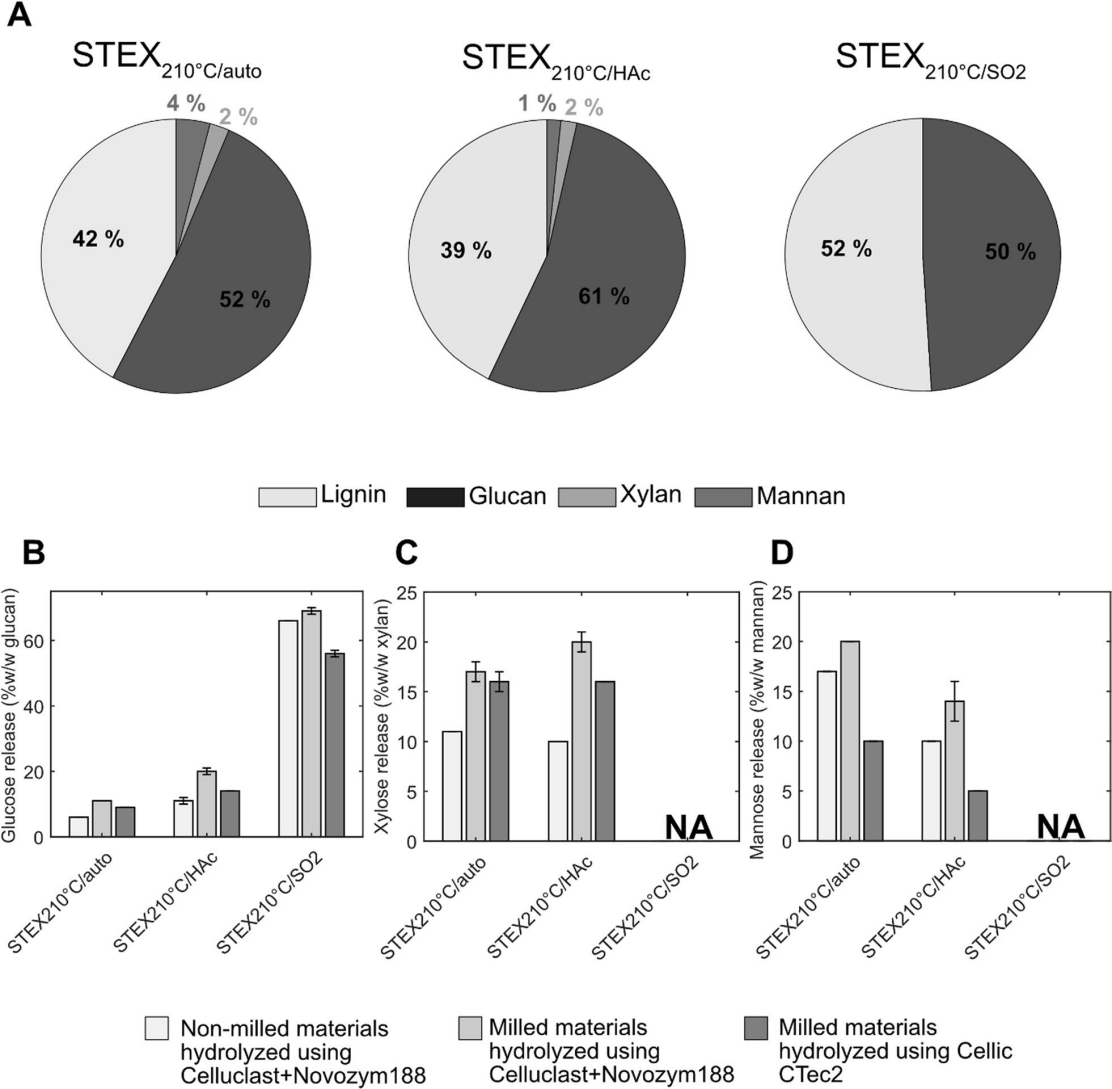
Materials composition and comparison of sugar release from steam-pretreated spruce, with or without an additional knife-milling step. **A** Materials composition expressed as percentage dry mass (*w*/*w*). **B**–**D** Release of **B** glucose, **C** xylose, and **D** mannose was measured after 48 h of hydrolysis. Please note that glucose can be released from both cellulose and galactoglucomannan. Data represent mean values ± standard deviation of triplicate experiments. Where error bars are not represented, they are below 0.5%

Steam pretreatment was previously performed under three different conditions [13], resulting in two materials retaining hemicellulose (STEX_210°C/auto_ and STEX_210°C/HAc_) and one devoid of hemicellulose (STEX_210°C/SO2_). Loss of arabinose from arabinoglucuronoxylan and galactose from galactoglucomannan was observed in all three materials after pretreatment. The overall hemicellulose content for STEX_210°C/auto_, STEX_210°C/HAc_, and STEX_210°C/SO2_ was 6%, 3%, and 0% (*w*/*w*) dry matter, respectively (Fig. 1A).

The hydrolyzability of steam-pretreated spruce was assessed after incubation with Celluclast + Novozym 188 for 48 h (Fig. 1B–D). The hydrolysis efficiency of cellulose (measured as glucose) release from STEX_210°C/auto_, STEX_210°C/HAc_, and STEX_210°C/SO2_ resulted in 6%, 11%, and 66% (*w*/*w*) glucan, respectively (Fig. 1B).

To potentially increase enzyme accessibility, the materials were additionally milled after STEX and prior to enzymatic hydrolysis. As compared to the base case, where the materials were not milled, these yields represent increases in glucose release of 1.8-, 1.8-, and 1.0-fold for STEX_210°C/auto_, STEX_210°C/HAc_, and STEX_210°C/SO2_, respectively (Fig. 1B). A similar trend was observed for xylose and mannose, whose release improved by 1.6- and 1.2-fold with STEX_210°C/auto_ and by 2.0- and 1.4-fold with STEX_210°C/HAc_ following milling (Fig. 1C, D).

Increased hydrolysis efficiency after milling [39] has been linked to a reduction in particle size and, consequently, larger surface area [17]. However, due to the high energy demand, milling has an adverse impact on the process economy. Further, as most of the energy required dissipates into heat [16, 17], moisture content in the material can decrease. Hornification reactions may follow from the drying that to a varying degree follow milling. Hornification in turn may induce the cellulose ultrastructure to collapse [7, 40], further reducing cellulose accessibility to enzymes, as well as decreasing hydrolysis yields and rates [7].

In the present study, the impact of milling on hydrolysis yields was assessed on STEX_210°C/auto_, STEX_210°C/HAc_, and STEX_210°C/SO2_. Surprisingly, hydrolysis yields were lower with LPMO-containing Cellic CTec2 than Celluclast + Novozym 188 (Fig. 1). This was especially surprising as the LPMOs in Cellic CTec2 were expected to result in increased cellulose conversion yields. Such result could be explained by hydrolysis conditions being unsuitable for LPMOs. To ensure that LPMO can be active under the presented reaction conditions, various reductant and aeration conditions were tested in the next step. Because milling negatively affects the process economy, the following experiments were conducted using non-milled steam-pretreated material.

### Effect of reductant addition and oxygen availability on saccharification of steam-pretreated spruce by LPMO

The lower saccharification efficiency of Cellic CTec2 compared to Celluclast + Novozym 188, prompted the optimization of reaction conditions to ensure LPMO activity.

To assess LPMO activity, we measured the release of C4-oxidized cellobiose, known as Glc4gemGlc [41, 42]. Glc4gemGlc concentration was significantly higher (approximately 87%) in reactions run with Cellic CTec2 than Celluclast + Novozym 188 (Additional file 1: Fig. S1). This result confirmed LPMO activity in Cellic CTec2, but its near absent from Celluclast + Novozym 188.

**Fig. 2:**
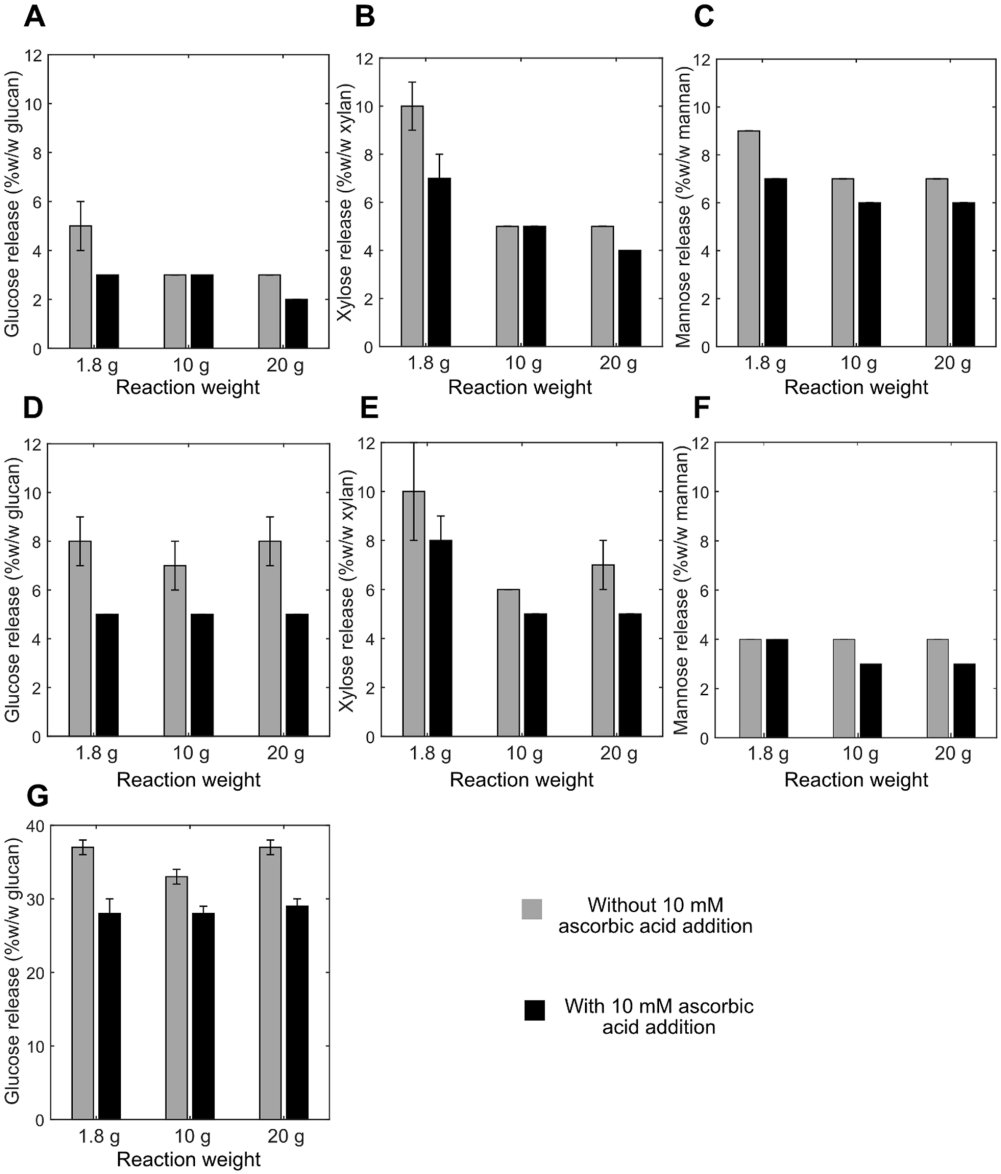
Influence of aeration and reductant supplementation on enzymatic hydrolysis yields using Cellic CTec2. Enzymatic hydrolysis of **A**–**C** STEX_210°C/auto_, **D**–**F** STEX_210°C/HAc_, and **G** STEX_210°C/SO2_ with or without 10 mM ascorbic acid. Different final reaction weights (1.8, 10, and 20 g) corresponding to different headspace volumes (10%, 80%, and 60%, respectively) of the total reaction volume were assessed. Release of **A**, **D**, **G** glucose; **B**, **E** xylose; and **C**, **F** mannose after 48 h of hydrolysis. Data represent mean values ± standard deviation of triplicate experiments. Please note that the scales have been adjusted for clarity. Where error bars are not represented, they are below 0.5%

To check if LPMO activity could be improved, reactions were supplemented with ascorbic acid as a reducing agent and aeration was increased by ensuring a larger headspace volume. Lignin has been shown to be a natural LPMO reductant, but the type and chemical state of lignin likely impacts its reducing power [18, 41, 43]. Different headspace volumes (10%, 60%, and 80%) were tested to augment O_2_ transfer and, hence, the supply of O_2_ as co-substrate for LPMO. In the presence of reductants (and transition metals), improved oxygenation can supply the enzyme with H_2_O_2_, another potential co-substrate [29]. The impact of reductant addition and increased aeration was assessed based on glucose, xylose, mannose, and Glc4gemGlc release (Fig. 2 and Additional file 1: Fig. S2). The results have been statistically analyzed using the Wilcoxon rank-sum test. Anaerobic conditions were not included into the study due to the need of H_2_O_2_ supplementation while we wanted to perform the enzymatic hydrolysis with minimal chemical additions.

When testing different headspace volumes, the oxidized glucose dimer Glc4gemGlc could be detected in almost all reactions (Additional file 1: Fig. S2), but without any improvement in sugar yields (Fig. 2). This finding indicated that the O_2_ available in the headspace of the smaller reaction tested (1.8 g and 10% headspace) was sufficient for LPMOs to catalyze their reaction. Statistical analysis did not show any significant difference when the different reactions weights were compared between themselves.

Interestingly, supplementation with ascorbic acid caused generally lower saccharification yields compared to lignin as sole reductant (Fig. 2). These results suggested that the lignin present in all three materials provided sufficient reducing power for LPMOs. In fact, ascorbic acid might have enhanced production of H_2_O_2_ from O_2_ inside redox reactions [27], which can cause auto-inactivation of LPMOs and also harm other enzymes in the cocktail [29, 38, 41]. It was not possible to use the Glc4gemGlc assay to analyze LPMO activity following ascorbic acid addition, likely due to interference from unknown compounds formed in the reaction (data not shown). Even though it was not possible to detect the Glc4gemGlc, we believe that the LPMOs have been activated during the enzymatic hydrolysis due to the use of similar reaction conditions employed in the LPMOs activity test (Additional file 1: Fig. S1). Similar conclusions were reached by Hansen et al. [45] in a study on Norway spruce previously steam-pretreated using 2-naphthol as a carbocation scavenger. This and the current work, indicate that lignin has sufficient reducing power to activate LPMO even in materials pretreated under the harshest conditions (e.g., STEX_210°C/SO2_). Such finding is surprising as the changes induced to the lignin during pretreatment, especially the loss of reactive bonds (e.g., beta-O-4) by condensation reactions, can be profound, as discussed in our previous study [13].

### Role of *Ta*LPMO9A in the hydrolysis of mildly pretreated spruce

**Fig. 3:**
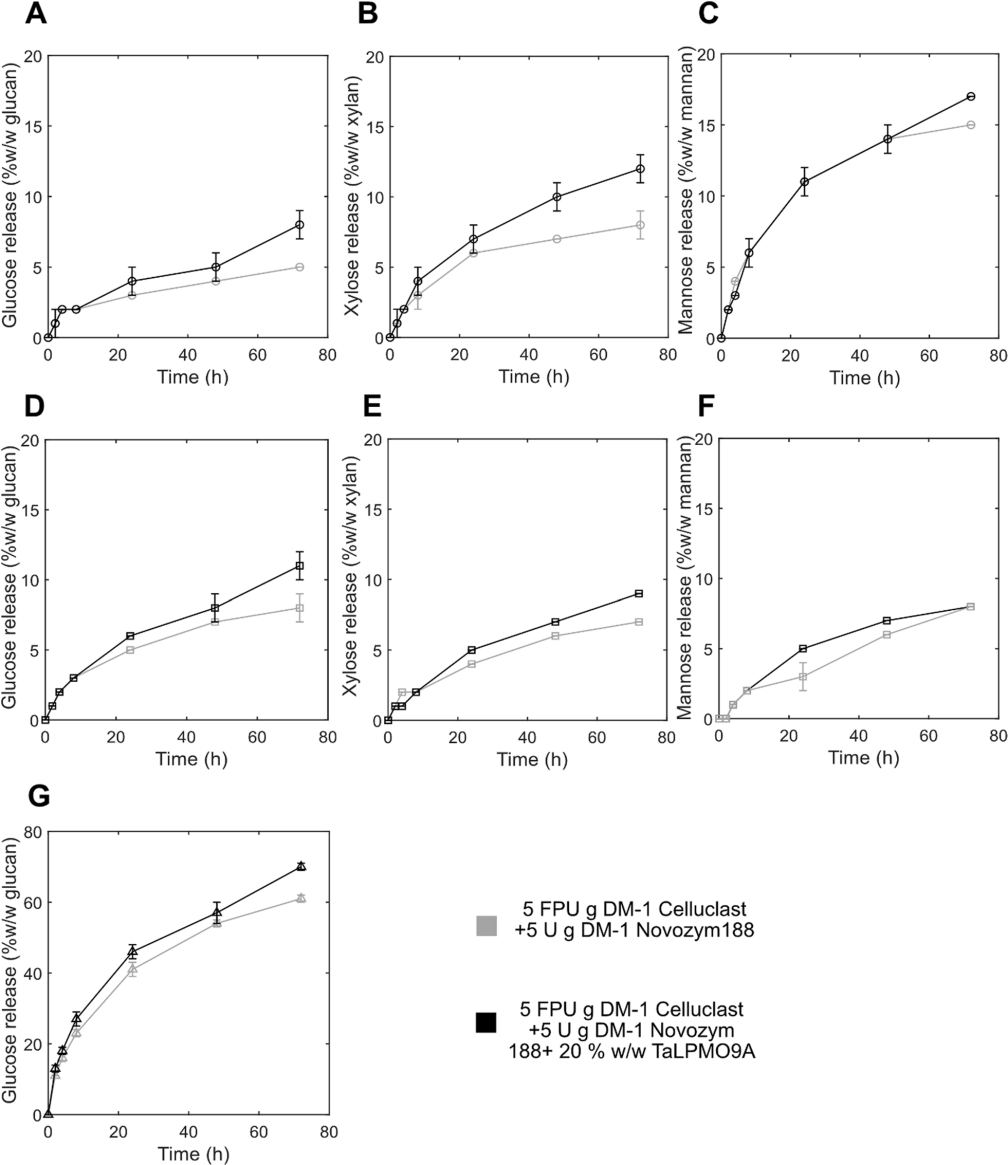
Time-course analysis of sugars released during enzymatic hydrolysis of steam-pretreated spruce with or without *Ta*LPMO9A supplementation. Enzymatic hydrolysis of **A**–**C** STEX_210°C/auto_, **D**–**F** STEX_210°C/HAc_, and **G** STEX_210°C/SO2_ with or without *Ta*LPMO9A. Release of **A**, **D**, **G** glucose; **B**, **E** xylose; and **C**, **F** mannose during 72 h of hydrolysis. Data represent mean values ± standard deviation of triplicate measurements. Please note that scales were adjusted for clarity. Where error bars are not represented, they are below 0.5%

**Fig. 4:**
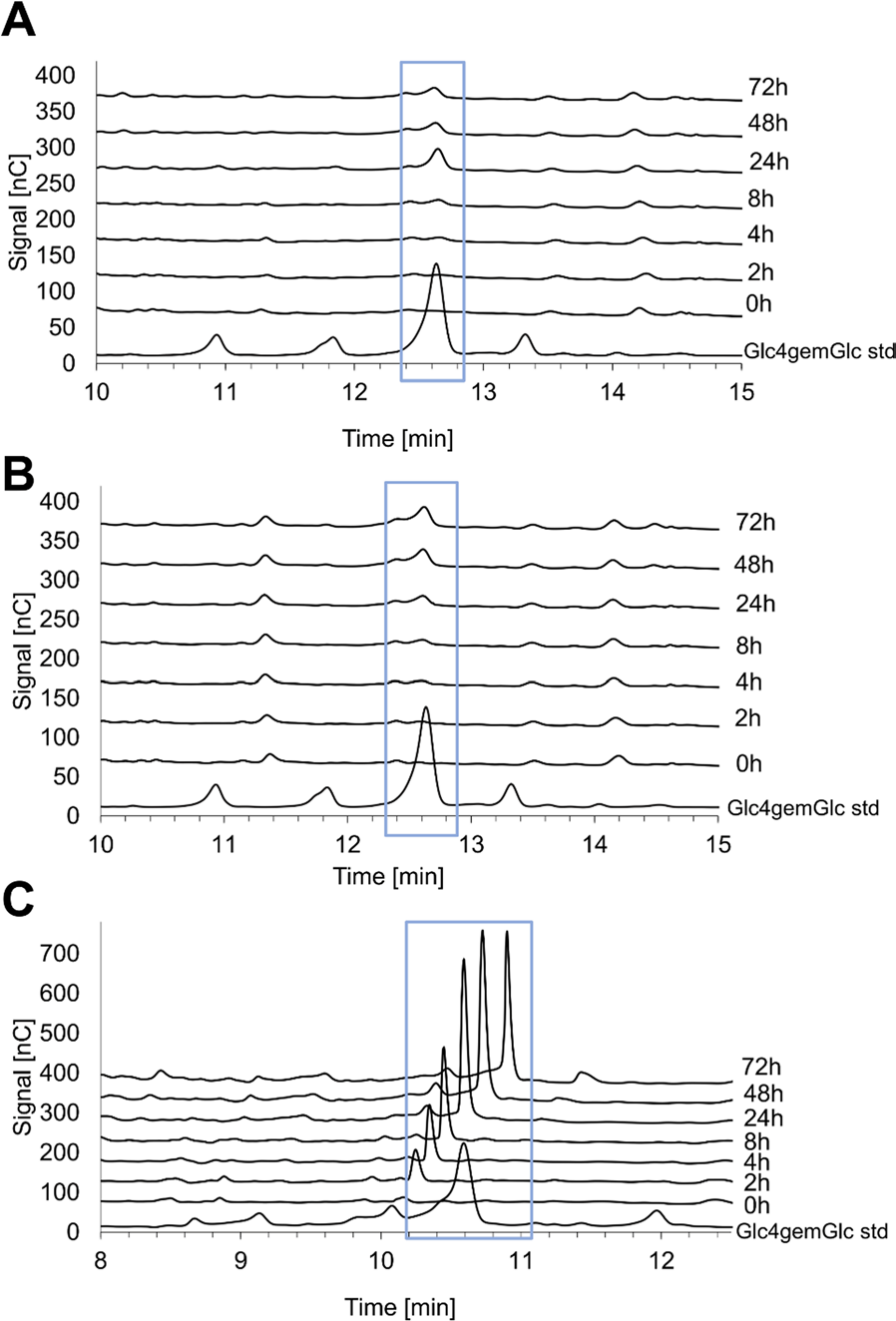
HPAEC-PAD chromatograms of the C4-oxidized glucose dimer Glc4gemGlc in time-course reactions with Celluclast + Novozym 188 supplemented with *Ta*LPMO9A. Chromatograms for **A** STEX_210°C/auto_, **B** STEX_210°C/HAc_, and **C** STEX_210°C/SO2_ (**C**). The reactions were run in triplicates. Given the elevated similarity between all triplicate chromatograms, only one of them is presented in the figure

Next, the effect of *Ta*LPMO9A on the saccharification of steam-pretreated spruce (STEX_210°C/auto_, STEX_210°C/HAc_, and STEX_210°C/SO2_) by Celluclast + Novozym 188 was investigated. As in the previous experiment, the release of glucose, xylose and mannose and Glc4gemGlc analysis (Fig. 3 and Fig. 4) were used to evaluate saccharification efficiencies and *Ta*LPMO9A activity, respectively. In addition, a time course of the release was plotted to gain insight on the role of LPMO during different phases of enzymatic hydrolysis.

*Ta*LPMO9A supplementation achieved consistently higher glucose release compared to Celluclast + Novozym 188 alone, with 1.6-, 1.4-, and 1.1-fold greater values for STEX_210°C/auto_, STEX_210°C/HAc_, and STEX_210°C/SO2_, respectively (Fig. 3). Using STEX_210°C/auto_ as substrate, Glc4gemGlc started to accumulate within 4–8 h, rose until 24 h (Fig. 4A), and decreased thereafter. A similar effect was observed previously [41], and was ascribed to abiotic (on-column) degradation of Glc4gemGlc at the high pH encountered during analysis. When STEX_210°C/HAc_ was the substrate, Glc4gemGlc accumulation started again within 4–8 h, but then increased until 48 h (Fig. 4B). The delayed onset of Glc4gemGlc detection does not necessarily indicate inactive LPMOs. The enzyme likely started working on the cellulose, creating new oxidized ends in the glucan chain, but Glc4gemGlc could not yet be revealed by high-performance anion-exchange chromatography with pulsed amperometric detection (HPAEC-PAD). Finally, using STEX_210°C/SO2_ as substrate (Fig. 4C), Glc4gemGlc started to accumulate already within 0–2 h and increased until 24 h.

A comparison of the sugar release time course (Fig. 3) with Glc4gemGlc development (Fig. 4), gives additional insights into the role of *Ta*LPMO9A in softwood saccharification. It seems that the beneficial impact of *Ta*LPMO9A supplementation on glucose release only became apparent in the later part of hydrolysis. In fact, the initial rates of hydrolysis were similar for non-supplemented and *Ta*LPMO9A-supplemented reactions (Fig. 4). LPMOs are believed to aid cellulose saccharification by creating nicks in the glucan chain on the more ordered (crystalline) parts of the cellulose microfibril. These nicks provide new binding sites for processive hydrolytic cellulases, which can then start degrading the cellulose by surface erosion*.* Due to the greater energy required to hydrolyze crystalline cellulose [46], the rate is significantly lower compared to that of less ordered or para-crystalline cellulose [7]. This might explain why the positive effect of *Ta*LPMO9A becomes apparent only in the later stages of hydrolysis, as the slower processive enzymes make use of the nicks created at the beginning of the reaction. As reported in ‘‘Ultrastructural characterization of cellulose before and after enzymatic hydrolysis’’ Section, crystallinity (i.e., ratio of highly ordered to less ordered cellulose) increased with increasing pretreatment severity (from STEX_210°C/auto_ to STEX_210°C/SO2_). The higher crystallinity of STEX_210°C/SO2_ might have resulted in a slower hydrolysis rate, possibly explaining the lesser effect of *Ta*LPMO9A compared to mildly pretreated STEX_210°C/auto_ and STEX_210°C/HAc_.

Additionally, *Ta*LPMO9A supplementation led to a clear improvement in xylose release (Fig. 3B–E) in mildly pretreated STEX_210°C/auto_ and STEX_210°C/HAc_ (1.5- and 1.3-fold, respectively). In a previous study, we showed that the residual hemicellulose is highly recalcitrant towards hydrolysis due to its low amounts and the presence of lignin–carbohydrate complexes [13]. Separately, we reported that *Thermothielavioides terrestris* LPMO9 could be active on arabinoglucuronoxylan from spruce [32]. Collectively, these findings suggest that *Ta*LPMO9A could promote the hydrolysis of the xylan backbone. Specifically, *Ta*LPMO9A might act in concert with enzyme cocktails, increasing xylose release and facilitating cellulose hydrolysis by removing hemicellulose. This, in turn, increases enzyme accessibility.

### Ultrastructural characterization of cellulose before and after enzymatic hydrolysis

To further elucidate the role of *Ta*LPMO9A in saccharification of STEX_210°C/auto,_ STEX_210°C/Hac_, and STEX_210°C/SO2_, changes to cellulose ultrastructure were analyzed by WAXS before and after enzymatic hydrolysis using Celluclast + Novozym 188, with or without *Ta*LPMO9A addition. The presence of crystalline and para-crystalline cellulose domains led to a distinct scattering pattern (Additional file 1: Figs. S3, S4), which was used to determine relative crystallinity as well as the size of cellulose crystallites. The width of the crystallite determined from the 200 crystalline plane is often related to the width of cellulose microfibrils [47].

**Fig. 5:**
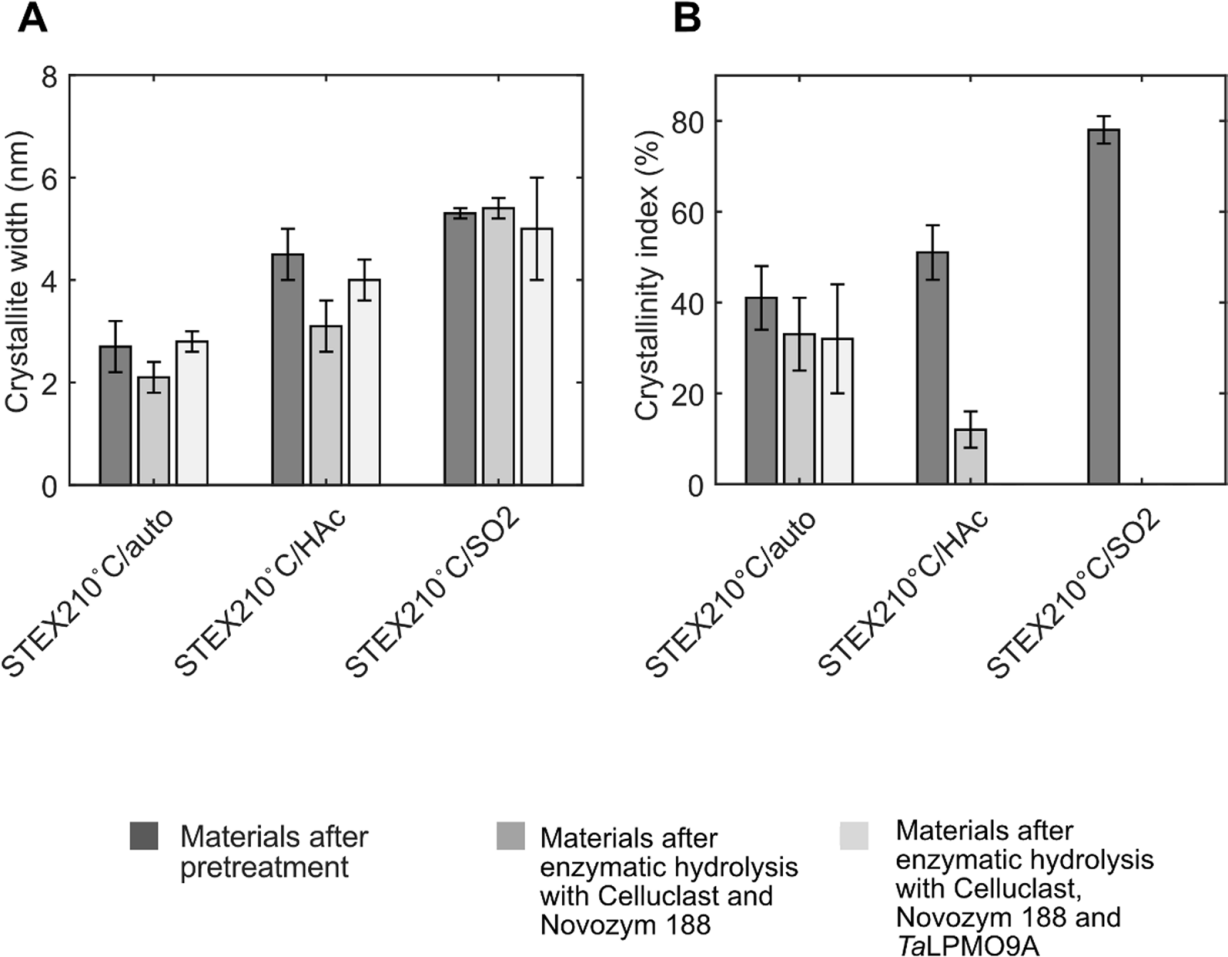
Crystallite size (**A**) and crystallinity index (**B**) of steam-pretreated spruce samples before and after enzymatic hydrolysis, with or without *Ta*LPMO9A, determined by WAXS analysis

As shown in a previous study [13], increased pretreatment severity resulted in greater crystallite width (Fig. 5A). This can be explained by the removal of less ordered cellulose and hemicellulose, with consequent aggregation of cellulose microfibrils.

Here, enzymatic hydrolysis did not affect crystallite width but, instead, drastically reduced cellulose crystallinity (Fig. 5). Considering that hydrolysis of crystalline cellulose proceeds as surface erosion by processive cellulases [46], a decrease in crystallite width was expected. A possible explanation is that, by the end point of hydrolysis (Fig. 5A), fiber areas that were accessible to the enzymes were degraded completely, but the more crystalline domains were unaltered and, hence, did not decrease in size.

Next, cellulose crystallinity before and after enzymatic hydrolysis was analyzed (Fig. 5B). No statistically significant changes in crystallinity were detected during hydrolysis of STEX_210°C/auto_. However, in the other two materials, the impact of the enzymatic hydrolysis on the cellulose crystallinity was pronounced. In the case of STEX_210°C/HAc_ with Celluclast + Novozym 188, the crystallinity index decreased from 51 to 12%, becoming negative when *Ta*LPMO9A was supplemented. Similar results were obtained with STEX_210°C/SO2_. A negative value of the crystallinity index indicates that the relative amount of crystalline regions dropped below that of less ordered domains (see Eq. 1). Collectively, WAXS data indicate that more severe pretreatment facilitates cellulose accessibility to enzymes (both hydrolytic and oxidative). More interestingly, the scattering data are a direct proof that supplementation with *Ta*LPMO9A promotes a decrease in cellulose crystallinity, most likely owing to the enzyme’s direct (i.e., oxidative degradation) and indirect (i.e., creating new access points for processive cellulose hydrolases) impact. In support of this mechanistic hypothesis, the largest drop in crystallinity was observed with material subjected to the harshest pretreatment conditions (STEX_210°C/SO2_), which coincided also with the highest Glc4gemGlc release (Fig. 4).

## Conclusions

In the present study, we investigated the role of LPMO9 in the saccharification of differentially pretreated softwood substrates. Results suggest that the requirements of LPMOs for O_2_ as co-substrate and reductant are met, respectively, by aeration over a relatively small headspace and the presence of lignin in the softwood substrates. Although *Ta*LPMO9 was active on all three softwood substrates, the highest Glc4gemGlc release was found in the material subjected to the harshest pretreatment (STEX_210°C/SO2_), along with a more pronounced drop in cellulose crystallinity. Taken together, these results indicate that the enzyme benefitted from easier accessibility to cellulose created by the harsher pretreatment. Time-course analysis of *Ta*LPMO9-supplemented reactions showed that the time frame of Glc4gemGlc accumulation was substrate-dependent. The beneficial impact of LPMOs manifests itself only in the later stages of hydrolysis, likely because processive cellulose hydrolases need some time to exploit the newly created access points. The findings in this study provide new insights on the role and practical applicability of LPMOs in the saccharification of softwood substrates. Accordingly, the use of LPMOs and steam pretreatment speed up the fractionation of spruce and improve monosaccharide yields.

## Materials and methods

### Steam-pretreated spruce and compositional analysis

Milled Norway spruce (*Picea abies*) chips were kindly provided by the Widtskövle sawmill. Steam pretreatment was performed as described elsewhere [13]. The conditions used for the pretreatment, as well as the combined severity factor [48] are reported in Table 1. Briefly, the conditions were 210 °C for 5 min, with/without addition of an acid catalyst, such as 1% (*w/w*) acetic acid (HAc) or 3% (*w/w*) SO_2_. Carbohydrate composition of steam-pretreated materials was analyzed following the National Renewable Energy Laboratory/TP-510–42,618 protocol [49].

**Table 1:** Pretreatment conditions of softwood used in this study

	STEX_210°C/auto_	STEX_210°C/HAc_	STEX_210°C/SO2_
Temperature (°C)	210	210	210
Catalyst (% *w/w*)	Autocatalyzed	1% HAc	3% SO_2_
pH liquid fraction	3.6	3.1	1.5
CSF	1.0	1.5	3.1

^a^Materials pretreated in Caputo et al. 2022 [13]

*CSF* combined severity factor

### Activities measurements and protein quantification of enzymatic cocktails

Three enzyme cocktails (Celluclast, Novozym 188, and Cellic CTec2) were used for enzymatic hydrolysis; all were kindly provided by Novozymes A/S (Bagsværd, Denmark). The enzymes were loaded based on activity measurements. The cellulolytic activity in Celluclast and Cellic CTec2 was determined using the filter paper unit (FPU) assay [50] with some adjustments [51]. The β-glucosidase activity of Novozym 188 was determined as described previously [52] with some adjustments [13]. Protein concentration was measured with the Bradford method using bovine serum albumin to generate a calibration curve [53]. Prior protein precipitation was performed as described hereafter. For every 200 µL of sample, 20 µL of 500 mM K_3_PO_4_, 20 µL of 250 mM CaCl_2_, and 500 µL of pure ethanol were added. After mixing, the samples were centrifuged at 14,000 rpm for 1 min, the supernatant was removed, and the Bradford assay was performed on the dissolved pellet.

### Comparison of LPMO activity in the cellulolytic Celluclast + Novozym 188 and LPMO-containing Cellic CTec2 cocktails

To compare LPMO activity, we followed the release of Glc4gemGlc (C4-oxidized cellobiose) by Celluclast + Novozym 188 and Cellic CTec2 over time on Avicel. The reactions were set up in duplicate in 50 mL Falcon tubes with 10 mL of reaction volume. The tubes were placed in the incubator horizontally for optimal orbital mixing at 200 rpm. All reactions contained 10% (*w*/*w*) Avicel PH-101 (from cotton linters; Sigma Aldrich, St. Louis, MO, USA) and 4 mg enzyme/g dry mass (5:1 *w*/*w* for Celluclast + Novozym 188). The enzyme loadings of Celluclast, Novozym 188 and Ctec2 correspond to 0.9 FPU g^−1^ dry mass, 0.1 U g^−1^ dry mass and 17.5 FPU g^−1^ dry mass, respectively. The reactions were incubated in 0.1 M acetate buffer (pH 5) at 50 °C. Ascorbic acid was added at 1, 5, and 10 mM to activate LPMO, and samples were taken at 0, 2, 25, and 48 h. Control reactions contained either no enzymes or no reductant, and did not yield any Glc4gemGlc. All reactions were stopped by boiling for 15 min, followed by filtering through 0.45 µm filter plates (Millipore, Darmstadt, Germany). The samples were stored at − 20 °C and were diluted fourfold in MilliQ water prior to HPAEC-PAD analysis.

### Enzymatic hydrolysis of milled materials

The materials were milled for 30 s in an IKAA10S knife mill (IKA^®^-Werke GmbH and Co. KG, Staufen, Germany), after which the dry mass was measured. Enzymatic hydrolysis experiments were performed in triplicates, in 2-mL screw-cap tubes with a total reaction weight of 1.8 g. The loaded substrate (2% *w*/*w* of dry mass) was suspended in 0.15 M acetate buffer (pH 5). The reaction mixture was autoclaved at 121 °C for 20 min, after which the sterile-filtered enzyme cocktails were added aseptically at 10 FPU g^−1^ dry mass for Celluclast and 10 U g^−1^ dry mass for Novozym 188. The cellulolytic activity of Celluclast was 50 FPU mL^−1^, while the β-glucosidase activity in Novozym 188 was 20 U mL^−1^. Enzymatic hydrolysis was carried out at 40 °C for 48 h in an oven with end-over-end rotation (Big S.H.O.T III^™^; Boekel Scientific, Feasterville, PA, USA) with constant rotation at 25 rpm. To terminate the reaction, the mixture was boiled for 10 min. The samples were then either stored at − 20 °C or processed directly as detailed in ‘‘Analytical methods and data processing’’ Sect.

### Enzymatic hydrolysis with different headspaces and addition of ascorbic acid

Enzymatic hydrolysis experiments were performed in triplicates with total reaction weights of 1.8, 10 and 20 g. Hydrolysis with 1.8 g reaction weight was performed in 2 mL screw-cap tubes; whereas the latter two were performed in 50 mL tubes. The headspace volume corresponded to 10%, 80%, and 60% of the total reaction volume, respectively. As it was not possible to autoclave (121 °C for 20 min) the reactions assembled in 50 mL tubes, the material was autoclaved beforehand. After that, the dry mass was measured and the loaded substrate (2% *w*/*w* of dry mass) was suspended in 0.15 M acetate buffer (pH 5). Enzymatic hydrolysis was carried out as detailed in ‘‘Enzymatic hydrolysis of milled materials’’ Sect. Sterile-filtered Cellic CTec2 was added at 10 FPU g^−1^ dry mass, with measured cellulolytic activity of 140 FPU mL^−1^. To terminate the reaction, the mixture was boiled for 10 min. The samples were then either stored at − 20 °C or processed directly as detailed in ‘‘Analytical methods and data processing’’ Sect.

### Time-course reactions

Time-course reactions were performed in triplicates for each time point (0, 2, 4, 8, 24, 48, and 72 h), in 2 mL screw-cap tubes with a total reaction weight of 1.8 g. The loaded substrate (2% *w*/*w* of dry mass) was suspended in 0.15 M acetate buffer (pH 5). The reaction mixture was autoclaved at 121 °C for 20 min, after which sterile-filtered enzyme cocktails were added aseptically at 5 FPU g^−1^ dry mass (Celluclast) and 5 U g^−1^ dry mass (Novozym 188). The cellulolytic activity in Celluclast was 53 FPU mL^−1^ while the β-glucosidase activity in Novozym 188 was 30 U mL^−1^. *Ta*LPMO9A was supplemented on top of the enzyme cocktails at 1.9 mg protein g dry mass^−1^, which corresponded to 20% (*w*/*w*) of the protein content loaded in with Celluclast and Novozym 188. Protein content was measured as described in Sect. "Activities measurements and protein quantification of enzymatic cocktails", and amounted to 21.5 g L^−1^ (Celluclast) and 34.0 g L^−1^ (Novozym 188). Enzymatic hydrolysis was carried out as detailed in ‘‘Enzymatic hydrolysis of milled materials’’Sect. To terminate the reaction for the different time points, the mixture was boiled for 10 min. The samples were then either stored at − 20 °C or processed directly as detailed in the ‘‘Analytical methods and data processing’’ Sect.

*Ta*LPMO9A (UniProt ID G3XAP7) was kindly provided by Novozymes AS. The LPMO was Cu-saturated prior to setting up the reactions by first incubating the pure enzyme at a 1:3 molar ratio with CuSO_4_ for 30 min, followed by buffer exchange to 50 mM BisTris–HCl (pH 6.5) using 10 kDa molecular weight cut-off Macrosep centrifugal filters (PALL, Port Washington, NY, USA). *Ta*LPMO9A concentration was determined spectrophotometrically using a Nanodrop (Thermo Fisher Scientific, Waltham, MA, USA) and the theoretical extinction coefficient (45,630 M^−1^ cm^−1^) was calculated using the ProtParam tool (https://web.expasy.org).

### Wide-angle X-ray scattering

WAXS measurements were performed using a Mat: Nordic instrument (SAXSLAB, Copenhagen, Denmark) equipped with a MicroMax-003 + high brilliance microfocus Cu- radiation source (Rigaku, Tokyo, Japan) and a Pilatus 300 K detector (Dectris AG, Baden-Daettwil, Switzerland). X-ray wavelength was 1.54 Å^−1^ and the measured *q*-range was 0.07–2.7 Å^−1^ and 2.5–3.5 Å^−1^ with the secondary Pilatus 100 K detector. Before every new set of measurements, the *q*-axis was calibrated by measuring a silver behenate sample. Samples were measured at room temperature and sealed in sandwich cells covered with mica windows. The measurements were performed on three different pieces of each sample. The two-dimensional scattering patterns were radially averaged using SAXSGui software (SAXLAB), while Fityk software (https://fityk.nieto.pl) was used for background subtraction and peak deconvolution. The diffractograms were deconvoluted using Gaussian functions. In addition, cells containing Milli-Q water and soluble spruce hemicellulose (galactoglucomannan) samples extracted from spent-sulfite liquor were measured to model amorphous peaks. The amorphous region was modeled with two or three Gaussian functions. The diffractograms of galactoglucomannan in water are presented in Additional file 1: Fig. S5. Detailed chemical composition of the hemicellulose samples is presented elsewhere [54].

The crystallinity index was calculated according to Eq. 1 [55]:

1 $$Crystallinity\, index = 1-\frac{{I_{{{\text{amorphous}}}} }}{{I_{200} }},$$

where ${I}_{\mathrm{amorphous}}$ is the intensity of the amorphous peak (~ 19°) and ${I}_{002}$ is the intensity of the 200 reflection (~ 22.4°) [55]. The apparent crystallite size (*L*) was estimated using the Scherrer Eq. (2):

2 $$L = \frac{k\,\lambda }{{\beta \,\cos \Theta }},$$

where *K,* $\lambda , \beta ,$ and $\Theta$ represent the X-ray wavelength (1.542 Å), the half-height width of a diffraction peak, and the Bragg angle corresponding to the 200 crystalline plane, respectively. Their value in the equation is 0.94.

The positions of the peaks were first fixed to published values for 101 (15°), 10î (16.5°), 102 (20.4°), and 200 (22.4°) reflections, and were fitted once the appropriate fit was obtained. The 004 peak was not used due to poor data quality at higher angles.

### Analytical methods and data processing

Dry mass content was measured in triplicate as dry weight at 105 °C [56]. The release of glucose, mannose, and xylose was measured by isocratic HPAEC-PAD (ICS-5000; Dionex, Sunnyvale, CA, USA) using a Carbopac PA1 column as described elsewhere [57]. The samples were centrifuged for 5 min at 13,000 rpm, and the supernatants were filtered through 0.22-µm syringe-driven filters before storage at 4 °C until analysis.

The conversion of polymers to soluble monomeric sugars after enzymatic hydrolysis at low solids loading was calculated by Eq. 3 [58]:

3 $${Y}_{\left(g\right)}=\frac{{C}_{g}-{C}_{g0}}{{\varphi }_{G}{C}_{is0}{X}_{G0}},$$

where *Yg* is the conversion (expressed as % *w*/*w*) of cellulose to glucose (taken as example), *Cg* (g L^−1^) is the concentration of solubilized glucose in the sample supernatant, *Cg*_*0*_ (g L^−1^) is the initial glucose concentration, ${\varphi }_{G}$ is the molecular weight ratio of glucose-to-glucan monomer (*φ*_*G*_ = 180/162 = 1.11), *C*_*is0*_ (g L^−1^) is the initial concentration of insoluble solids, and *X*_*G0*_ is the initial mass fraction of glucan in insoluble solids.

Glc4gemGlc was analyzed by HPAEC-PAD on an IC-5000 instrument as described previously [32]. A Carbopac PA200 guard column (3 × 50 mm) and an analytical column (3 × 250 mm) from Dionex were used, and 10 µL of sample was injected. Duplicate or triplicate samples were analyzed. The Glc4gemGlc standard was prepared by incubating 2 µM *Nc*LPMO9C with 2 mM cellopentaose for 24 h at 200 rpm and 40 °C in 50 mM BisTris–HCl (pH 6.5). Ascorbic acid at a 2 mM final concentration was used to initiate the reaction. The reaction was stopped by boiling for 15 min and the mixture was stored at -20 °C. The reaction mixture was diluted 4- or 10-fold in MilliQ water prior to HPAEC-PAD analysis [22].

## Supplementary Information

**Additional file 1: Fig. S1.** Comparison of LPMO activity in Celluclast+Novozym 188 and Cellic CTec2 based on semi-quantification of the C4-oxidized glucose dimer Glc4gemGlc in a 25 h reaction on Avicel with 10 mm ascorbic acid as reductant. **Fig S2.** HPAEC-PAD chromatograms of Glc4gemGlc in 48 h reactions with Cellic CTec2, in which extra headspace was used to test the influence of aeration. **Fig S3.** Wide-angle X-ray scattering curves of steam-pretreated spruce samples after enzymatic hydrolysis without *Ta*LPMO9A. **Fig S4.** Wide-angle X-ray scattering curves of steam-pretreated spruce samples after enzymatic hydrolysis with *Ta*LPMO9A. **Fig S5.** Wide-angle X-ray scattering curves of a wet spruce galactoglucomannan sample.

## Data Availability

All data generated or analyzed during this study are included in this published article and its additional files.
