# Supplementary material for: Investigating the role of AA9 LPMOs in enzymatic hydrolysis of differentially steam-pretreated spruce
Source: Biotechnol Biofuels Bioprod. 2023 Apr 19;16:68. doi: 10.1186/s13068-023-02316-0 (PMC10114483; doi:10.1186/s13068-023-02316-0)
Supplement: Supplementary file 1 — Additional file 1: Fig. S1. Comparison of LPMO activity in Celluclast+Novozym 188 and Cellic CTec2 based on semi-quantification of the C4-oxidized glucose dimer Glc4gemGlc in a 25 h reaction on Avicel with 10 mm ascorbic acid as reductant. Fig S2. HPAEC-PAD chromatograms of Glc4gemGlc in 48 h reactions with Cellic CTec2, in which extra headspace was used to test the influence of aeration. Fig S3. Wide-angle X-ray scattering curves of steam-pretreated spruce samples after enzymatic hydrolysis without TaLPMO9A. Fig S4. Wide-angle X-ray scattering curves of steam-pretreated spruce samples after enzymatic hydrolysis with TaLPMO9A. Fig S5. Wide-angle X-ray scattering curves of a wet spruce galactoglucomannan sample. [file 13068_2023_2316_MOESM1_ESM.docx]

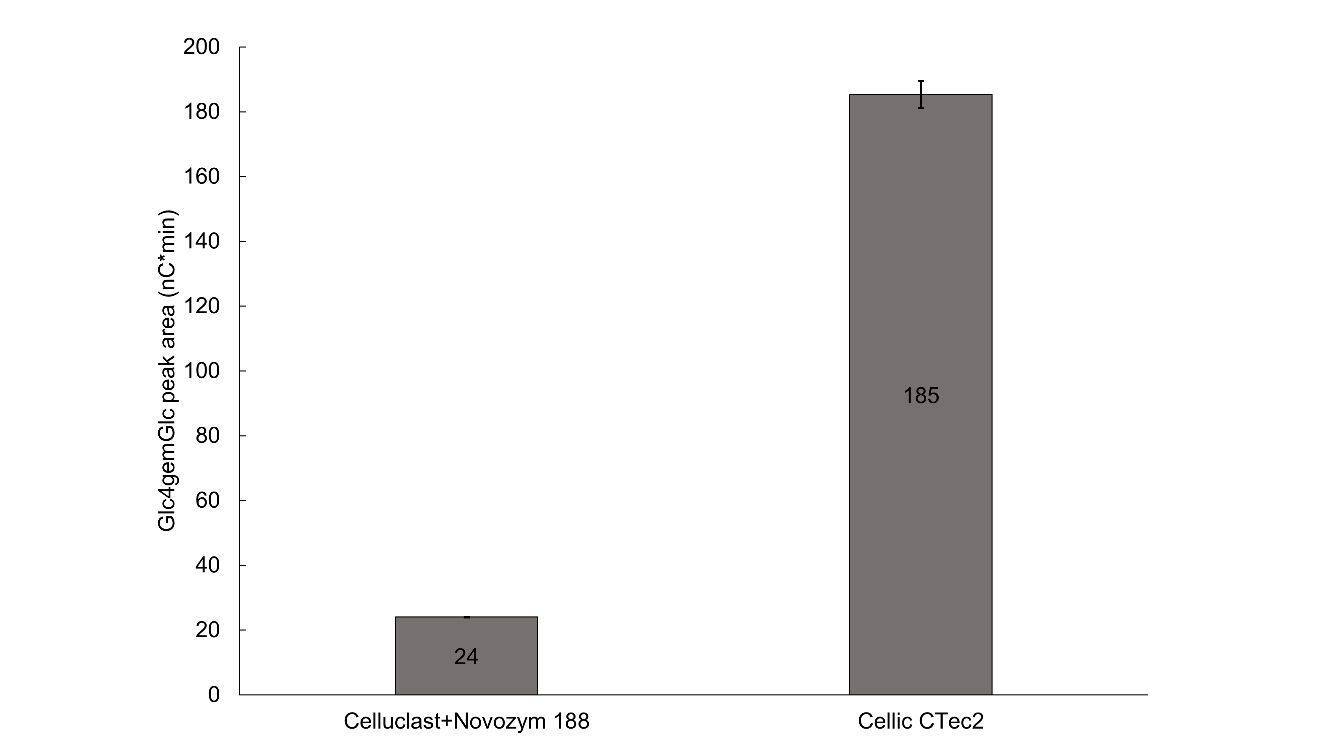


**Fig. S1 Comparison of LPMO activity in Celluclast+Novozym 188 and Cellic CTec2 based on semi-quantification of the C4-oxidized glucose dimer Glc4gemGlc in a 25-h reaction on Avicel with 10 mM ascorbic acid as reductant.** Reactions were carried out in a 10-mL reaction volume in 50-mL horizontally placed conical tubes subjected to orbital shaking at 50 °C and 200 rpm. Avicel concentration was 10% (w/w), enzyme loading was 4 mg/g dry mass (5:1 Celluclast:Novozym 188), and reactions were carried out in 100 mM acetate buffer (pH 5). Error bars represent the standard deviation of Glc4gemGlc peak areas of two independent reactions.


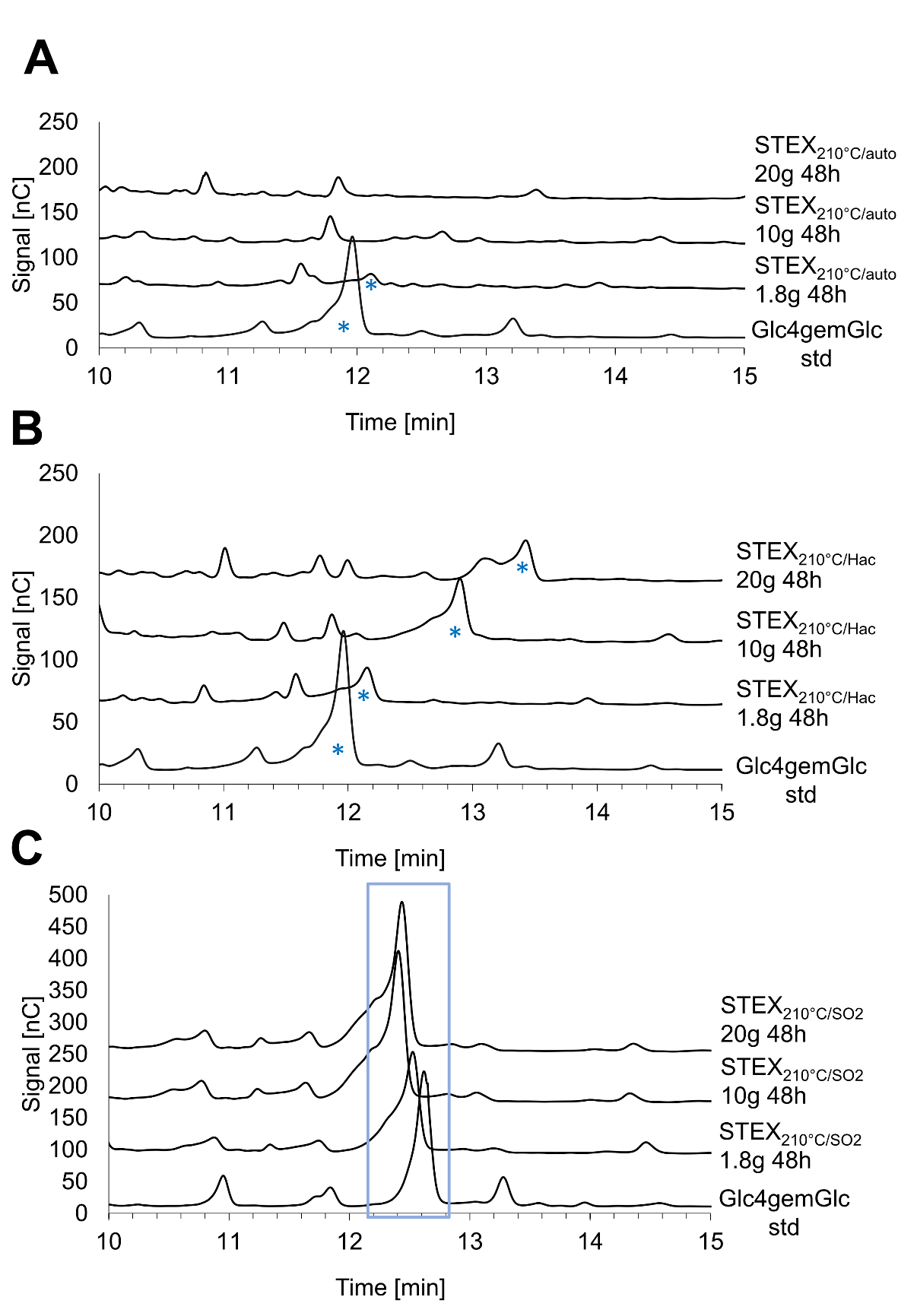


**Fig. S2. HPAEC-PAD chromatograms of Glc4gemGlc in 48-h reactions with Cellic CTec2, in which extra headspace was used to test the influence of aeration.** Reactions were carried out with (A) STEX_210°C/auto_, (B) STEX_210°C/HAc_, and (C) STEX_210°C/SO2_ as substrate. The 1.8 g reaction had the least headspace, the 10 g reaction had the most headspace, and the 20 g reaction had an intermediate headspace volume. The Glc4gemGlc peak is indicated with a blue asterisk or a blue box**.** As all triplicates were highly similar, only one of them is presented in the figure. Note that quantitative comparison of Glc4gemGlc release in the different headspace reactions was not possible under the tested conditions.


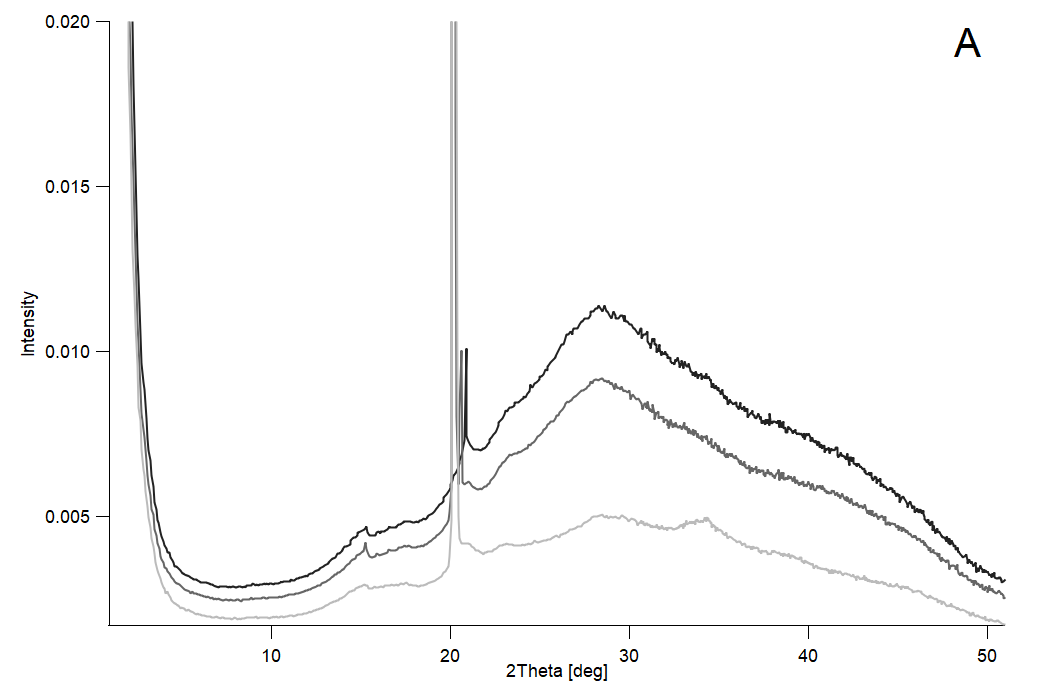

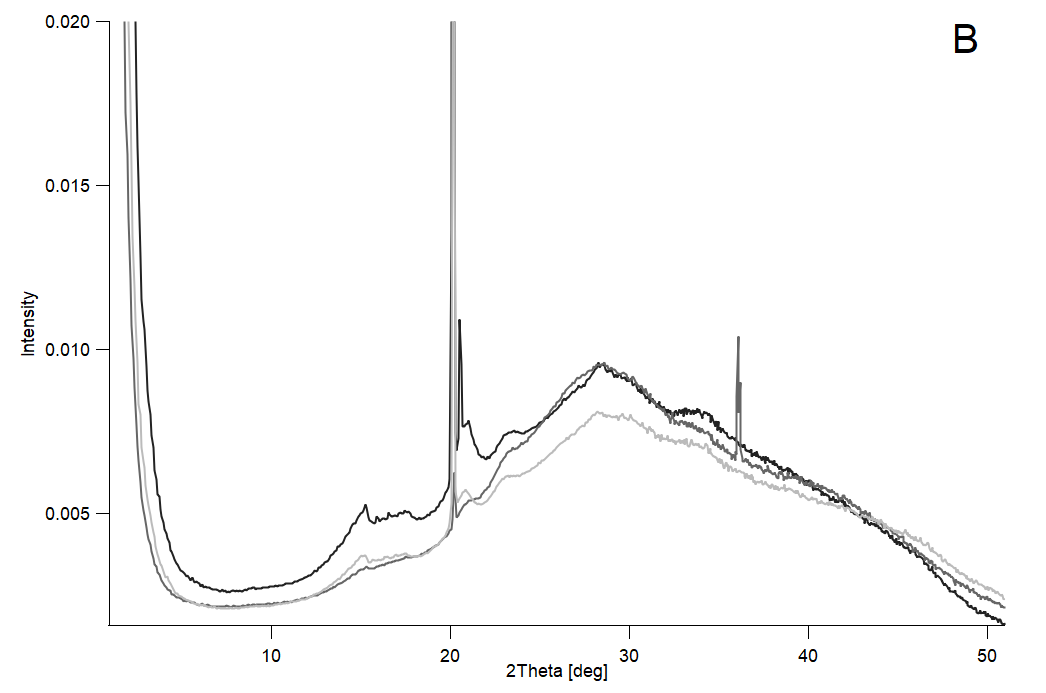

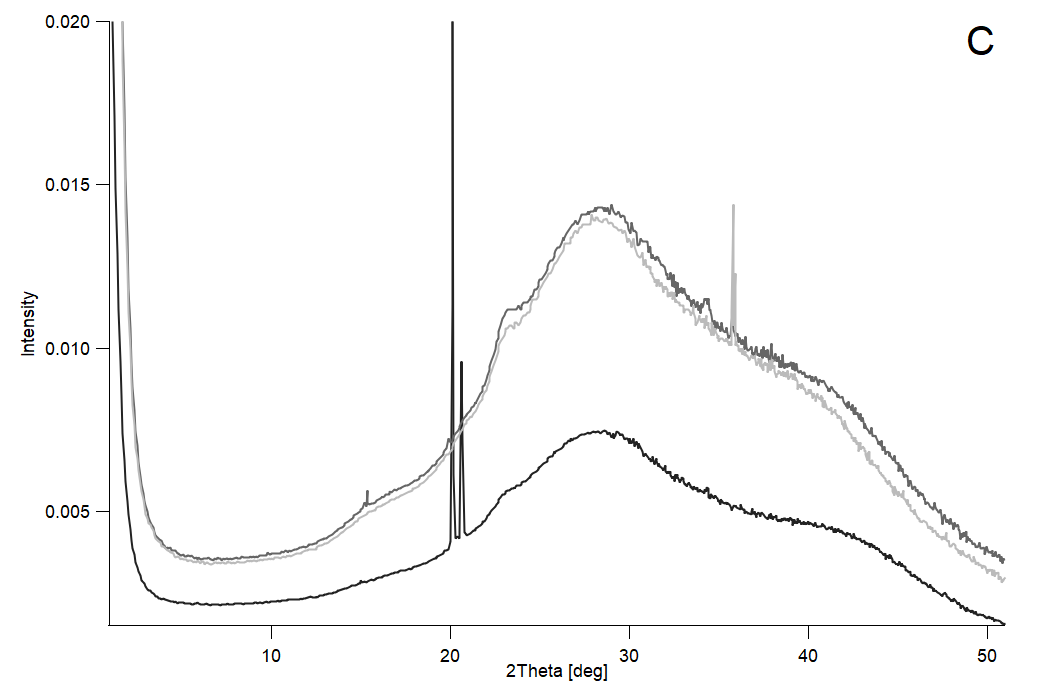


**Fig. S3. Wide-angle X-ray scattering curves of steam-pretreated spruce samples after enzymatic hydrolysis without *Ta*LPMO9A.** Scattering curves were obtained with (A) STEX_210°C/auto_, (B) STEX_210°C/HAc_, and (C) STEX_210°C/SO2_ as substrate.

**
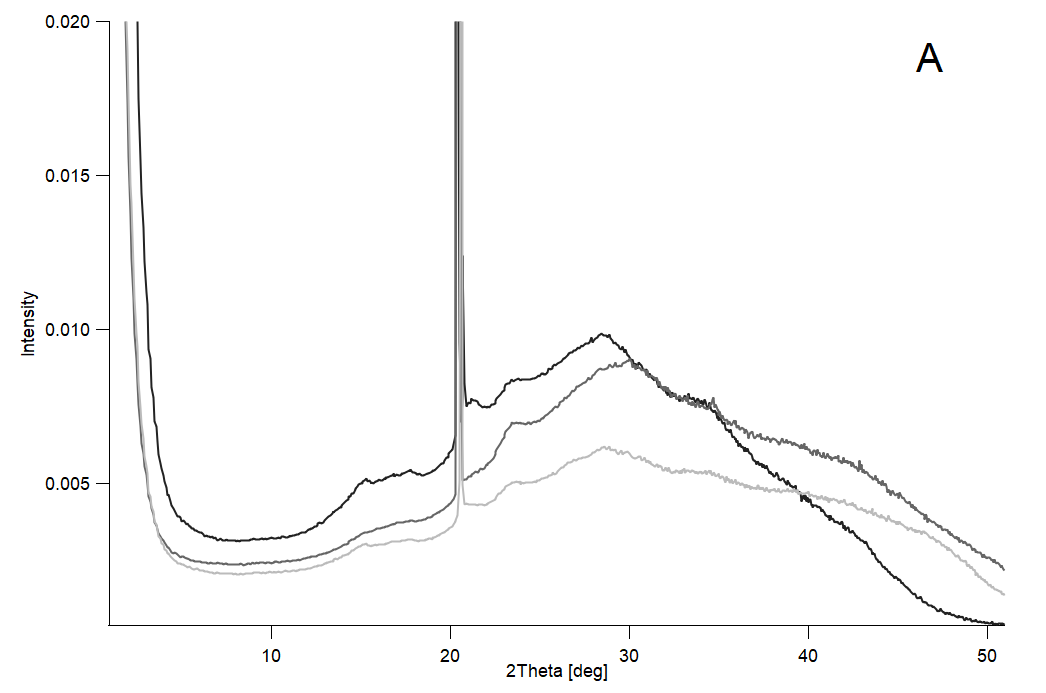

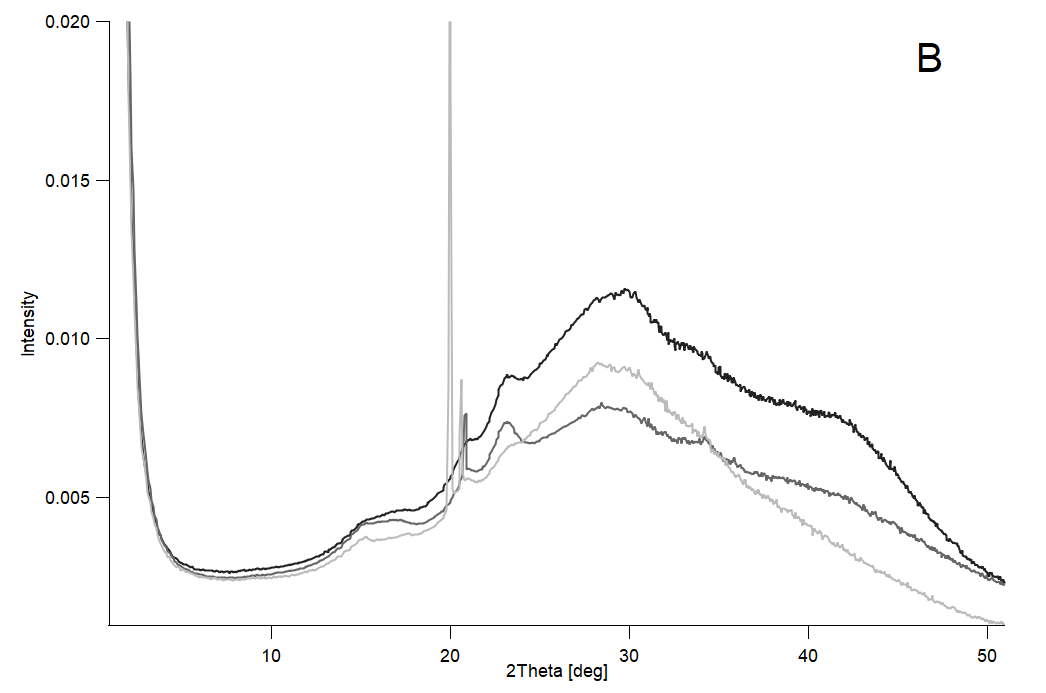

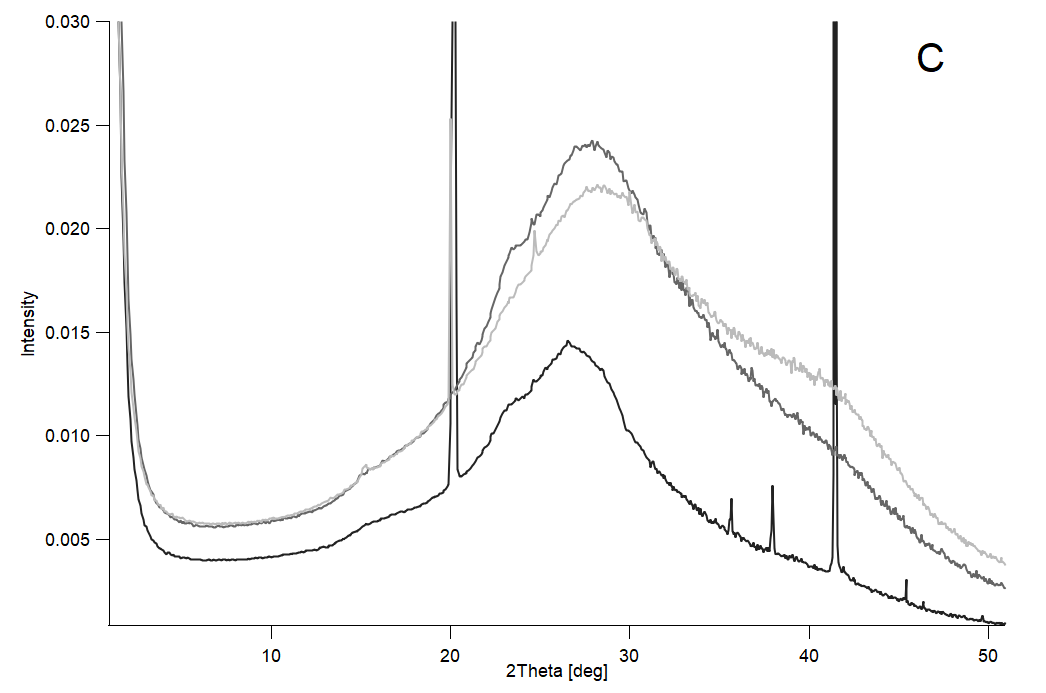
**

**Fig. S4. Wide-angle X-ray scattering curves of steam-pretreated spruce samples after enzymatic hydrolysis with *Ta*LPMO9A.** Scattering curves were obtained with (A) STEX_210°C/auto_, (B) STEX_210°C/HAc_, and (C) STEX_210°C/SO2_ as substrate.

**
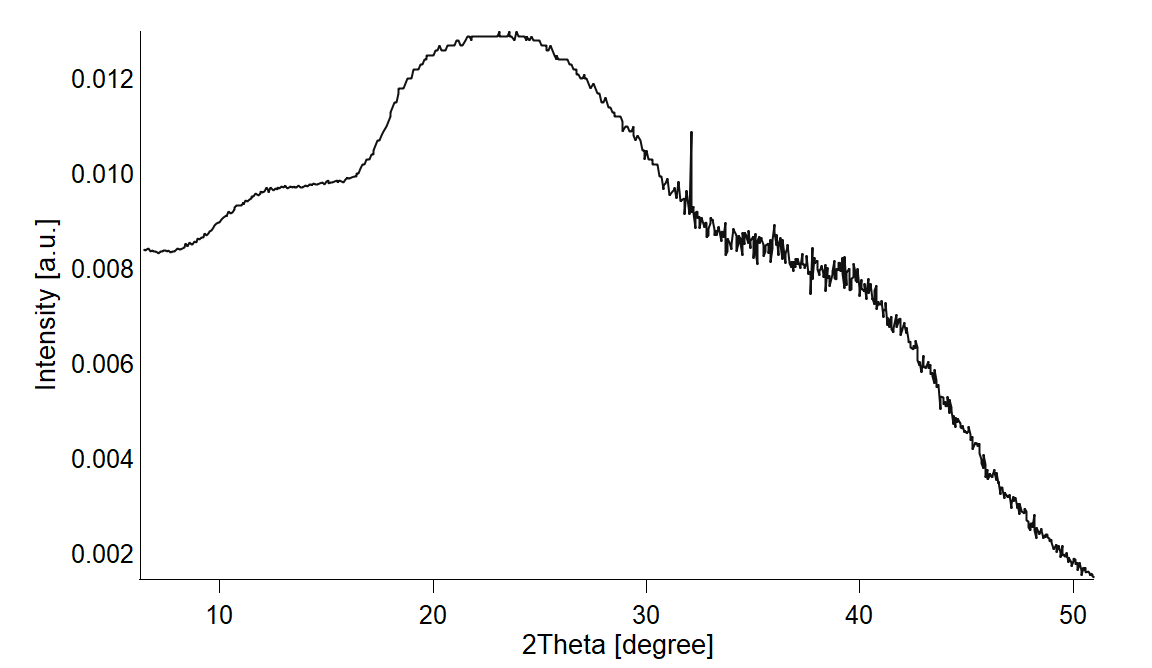
**

**Fig. S5. Wide-angle X-ray scattering curves of a wet spruce galactoglucomannan sample.**
